# Impact of HIV and antiretroviral drug exposure on lung growth and function over 2 years in an African Birth Cohort

**DOI:** 10.1097/QAD.0000000000002444

**Published:** 2019-11-11

**Authors:** Diane M. Gray, Catherine J. Wedderburn, Rae P. MacGinty, Lauren McMillan, Carvern Jacobs, Jacob A.M. Stadler, Graham L. Hall, Heather J. Zar

**Affiliations:** a Department of Paediatrics and Child Health, Red Cross War Memorial Children's Hospital and SA-MRC Unit on Child and Adolescent Health, University of Cape Town, Cape Town, South Africa; b Department of Clinical Research, London School of Hygiene & Tropical Medicine, London, UK; c Telethon Kids Institute and School of Physiotherapy and Exercise Science, Curtin University, Perth, Western Australia, Australia.

**Keywords:** antiretroviral therapy, HIV, HIV exposure, infant, lung function

## Abstract

**Objective:**

To assess the impact of HIV and antiretroviral exposure without infection on lung growth and function over the first 2 years of life.

**Design:**

Prospective observational study of an African birth cohort, Drakenstein Child Health Study.

**Method:**

Infants enrolled antenatally had lung function measured at 6 weeks, 1 and 2 years. HIV-infected women received antiretroviral therapy (ART) as per local guidelines. The association between HIV and antiretroviral exposure with lung function was assessed using mixed effects modelling.

**Results:**

Of 1143 infants born, two HIV-infected infants were excluded from analysis; 909 (80%) infants had lung function collected at 6 weeks [190 (21%) were HIV-exposed uninfected (HEU)]; 782 (69%) at 1 year and 741 (65%) at 2 years. At 6 weeks HEU infants had larger tidal volume compared with HIV-unexposed infants (1.13 ml, confidence interval: 0.02–2.23, *P* = 0.045). High maternal viral load was associated with a 17% lower expiratory flow over 2 years (0.17, confidence interval 0.00–0.34, *P* = 0.046). First-line ART initiated during pregnancy was associated with lower infant tidal volume at 6 weeks compared with those who initiated ART before pregnancy (−2.7 ml, −5.31 to −0.10, *P* = 0.042), and low maternal CD4^+^ cell counts associated with lower infant tidal over 2 years (−11.1 ml, −18.58–3.58, *P* = 0.004).

**Conclusion:**

HIV exposure is associated with altered lung function in early life, with a vulnerable HEU subgroup based on maternal disease severity, immunological compromise and ART exposure. These data highlight the importance of ongoing surveillance of respiratory health in HEU children.

## Abstract 2

Supplemental Digital Content is available in the text

## Background

Respiratory disease is the leading cause of death outside the neonatal period in children under 5 years worldwide [1] and despite progress towards the Sustainable Development Goals, many countries fall far behind the global under-5 mortality targets. This is particularly evident in sub-Saharan Africa (SSA) which has the highest under-5 mortality rate globally, and where children have a 15-fold higher likelihood of dying before 5 years compared with high-income countries [2]. In South Africa, despite progress reducing other causes of death including diarrhoea, little change has been made in the proportion of deaths attributable to pneumonia [3]. Understanding those factors which contribute towards paediatric respiratory disease in SSA is therefore critical to improving public health outcomes.

SSA is at the epicentre of the HIV/AIDS epidemic. The substantial reduction in perinatal transmission of HIV following the introduction of antiretroviral drugs is arguably the most significant success story of the epidemic; however, the number of HIV-infected women giving birth remains high, and has been reported as 1.4 million mothers each year for the past decade [4,5]. The decreasing rate of paediatric infections means that there is a growing population of uninfected children born following HIV and antiretroviral exposure, estimated around 15 million in 2017 [6]. HIV-exposed uninfected (HEU) children are recognized as having a greater risk of mortality and morbidity compared with unexposed children early in life [7,8]. In HEU children, respiratory disease is the primary cause of morbidity, with high rates of infectious disease [9]. Studies from South Africa have shown HEU infants have an increased risk of both bacterial infections including invasive pneumococcal disease [10] and viral infections such as respiratory syncytial virus (RSV) [11]. HIV exposure has also been found to be a key risk factor for pneumonia in infants [12,13].

In addition to global risk factors for respiratory disease in children such as poverty, maternal physical and psychological health, exposure to tobacco smoke and air pollution; HEU children experience additional risks through lower breast-feeding rates, living in HIV-affected households with higher rates of infectious diseases, exposure to altered immune environments *in utero* as well as to antiretrovirals [14]. Limited data are available on the impact of antiretrovirals; however, associations have been made with adverse birth outcomes including prematurity and low birth weight, and furthermore antiretrovirals have been hypothesized to affect lung growth and function through dysregulation of metabolic pathways [15]. However, improved maternal health through antiretroviral therapy (ART) initiation before pregnancy has been shown to reduce infectious morbidity and hospitalization in high-income countries [16].

Few studies have assessed lung function in SSA infants and few have focussed on HEU children globally. Novel early data from the Drakenstein Child Health Study (DCHS), a population-based birth cohort study in South Africa, showed differences in tidal volume aged 6 weeks between HEU and HIV-unexposed children [17]. HEU infants had higher tidal volumes on average compared with HIV-unexposed children, which may reflect an effect on breathing control. It is possible that the latter could be mediated through either exposure to HIV, or antiretrovirals taken by mothers and infants. An understanding of the long-term implications of HIV and antiretroviral exposure on uninfected child respiratory health in an SSA context is critically important.

In this analysis of the DCHS cohort, we aimed to assess the impact of HIV and antiretroviral exposure on lung growth and function over the first 2 years of life.

## Methods

### Study design and participants

This is a study of HIV-exposed uninfected and HIV-unexposed infants enrolled in the DCHS and who were followed from birth through to 2 years, with lung function measured at 6 weeks, 1 year and 2 years. The DCHS is a birth cohort study situated in a peri-urban, low socioeconomic area outside Cape Town in South Africa [18]. Mothers were enrolled antenatally between March 2012 and March 2015 and followed through pregnancy at one of two primary care clinics with mother–child pairs followed from birth. Infants attended scheduled study visits at 6, 10, 14 weeks and 6, 9 and 12 months of age and 6 monthly thereafter. In addition to these regular health assessments and monitoring, a strong surveillance system was established for the detection of lower respiratory tract illness (LRTI). Socioeconomic status was assessed as a composite variable, placing participants into relative quartiles. This score is derived from employment status and standardized scores of educational attainment, household income, assets and market access [19]. The study was approved by the Faculty of Health Sciences, Human Research Ethics Committee, University of Cape Town (401/2009; 423/2012) and by the Western Cape Provincial Health Research Committee. Parents gave informed, written consent in their first language for their infants to participate.

### HIV diagnosis and prevention of mother-to-child transmission

Maternal HIV infection was assessed at enrolment through self-report and routine prevention of mother-to-child transmission (PMTCT) HIV testing. All HIV-infected mothers received antiretroviral according to the Western Cape Department of Health Guidelines for PMTCT at the time. In 2012, the guidelines advised zidovudine (ZDV) in all pregnant women and ART to be initiated as per maternal clinical/immunological status. From early-2013 onwards the current guidelines were introduced which are triple ART irrespective of clinical status, made up of one nonnucleoside reverse transcriptase inhibitor and two nucleoside reverse transcriptase inhibitors [typically efavirenz (EFV) and tenofovir (TDF) and emtricitabine (FTC)/lamivudine] [20]. HIV data were obtained from folder reviews of mothers and children and accessing electronic laboratory data from the National Health Laboratory Service as well as self-report interviews antenatally and postnatally. In the case of multiple measures, the lowest recorded CD4^+^ cell count (collected 1 year before to 3 months after birth to maximise numbers) and highest viral load during pregnancy were used. HIV-exposed children were tested for HIV at 6 weeks (by PCR), 9 months (by PCR, ELISA or rapid antibody testing) and 18 months (by rapid antibody testing), as per provincial PMTCT guidelines.

### Lung function measures

Lung function testing was undertaken first at 6 (5–11) weeks of age corrected for prematurity (<37 weeks) and then at 1 year (11–13 months) and 2 years (23–25 months). All testing was done in unsedated, behaviourally assessed quiet sleep as previously described [21,22]. Lung function tests included measures of tidal breathing (tidal volume, respiratory rate, expiratory flow ratios) and sulphur-hexafluoride (SF6) multiple breath washout (MBW), which measures functional residual capacity (FRC) and the lung clearance index (LCI). The tidal lung volumes are a measure of lung growth. Low expiratory flow ratio [time to peak tidal expiratory flow over total expiratory time (*t*_PTEF_/*t*_E_)] reported here is associated with airway obstruction, though is also affected by lung size and breathing control [23]. The LCI is an early measure of small airways disease and impaired ventilation, it increases in disease states [24]. Measurements were collected using the Exhalyser D with ultrasonic flow meter (Ecomedics AG, Duernton, Switzerland) with 4% SF6 for the MBW [21].

### Statistical analysis

Analyses were conducted with STATA version 14.0 (College Station, Texas, USA). Descriptive data were presented as means, SDs and frequencies (proportions), as appropriate. Mann–Whitney rank sum tests was used to test for significant differences between categorical and continuous variables. Pearson chi-square test or Fisher Exact tests were used to determine if significant differences existed between categorical variables.

Lung function outcomes were modelled using linear regression to assess the impact of HIV exposure, maternal HIV disease severity and antiretroviral exposure on the lung function attained at each time point. Maternal HIV viral load was used as a categorical variable: undetectable (<40 copies/ml), detectable (≥40–1000 copies/ml) and virally unsuppressed (>1000 copies/ml). Maternal CD4^+^ cell count was categorized as more than 500, 350–500 and 350 cells/μl or less. BMI for age *z*-scores were calculated using the WHO Child Growth Standards ‘Igrowup’ STATA package.

Base models were first constructed using Directed Acyclic Graphs (DAGs) for confounder selection. DAGs minimal adjustment set of variables [socioeconomic status (SES), race, sex, BMI for age, maternal smoking] were used to assess the impact of exposures on lung function outcomes at each time point (6 weeks, 12 months and 24 months). In addition, mixed effects models were used to assess the impact of HIV and antiretroviral exposure on lung growth over 2 years. In this model LRTI episodes during 2 years was included given the documented impact of LRTI on lung function outcomes in this cohort [25]. For antiretroviral exposure, analyses were performed comparing all those children exposed to maternal triple ART, compared with ZDV, only. However, for timing of ART initiation we limited to the first-line regimen that the majority of women were receiving in the study (TDF/FTC/EFV) which is currently WHO recommended first-line in our setting (dolutegravir is not yet widely available), and by limiting to one regimen we were able to better examine the effects of timing without the confounding effects of different antiretroviral drugs. Estimated coefficients, 95% confidence intervals (CIs) and *P* values were recorded for each early life exposure of interest.

In addition, diagnostic checks were conducted. These included checking for normality in the residuals using histograms, standardized probability (*P*–*P*) plot, Quantile–Quantile (*Q*–*Q*) plots, as well the Shapiro–Wilk *W* test for normality. Further, homoscedasticity was checked using scatter plots and the presence of multicollinearity was explored using the variance inflation factor. Three of the lung function measures (FRC, ratio of time to peak tidal expiratory flow over total time of expiration and respiratory rate) were found to be nonnormal, and thus log-transformations were performed on these outcomes.

## Results

**Fig. 1:**
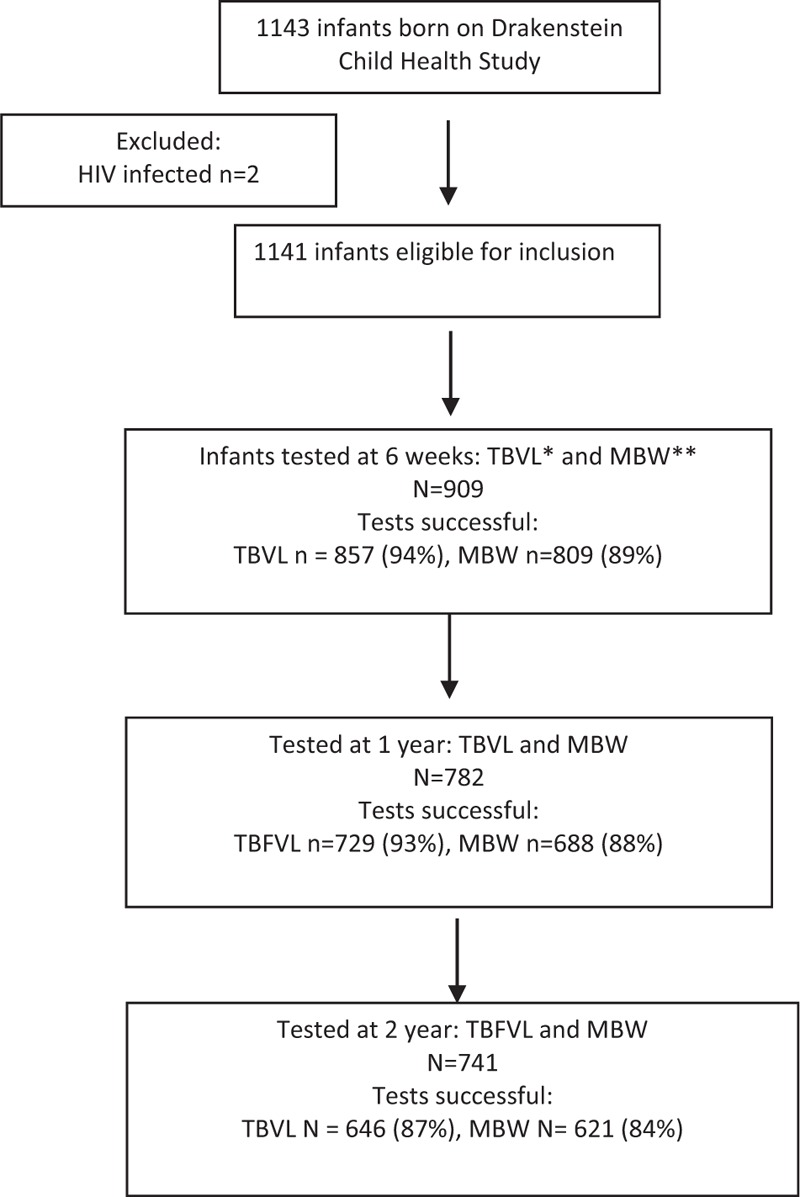
Flow chart of cohort (include number included at each time point with successful testing).

Due to the successful PMTCT programme only two of the 1143 infants born in the DCHS were HIV-infected, and these children were excluded from this analysis. Of the 1141 infants, 909 (80%) infants had lung function measured at 6 weeks; 190 (21%) were HEU; 473 (52%) male, 477 (53%) black African ancestry, 294 (33%) mothers smoked during pregnancy, 653 (72%) had household tobacco smoke exposure, Table 1. At 1 year 782 (69%) infants and at 2 years 741 (65%) infants had lung function measures collected, testing success rates shown in Fig. 1. HIV exposure prevalence was similar between those who were tested at 12 and 24 months and those who were not, Tables S1 and S2. The majority of HEU infants were black African (93 vs. 42% HIV-unexposed, *P* < 0.001), HEU infants had less household smoke exposure (68 vs. 81%, *P* < 0.001) and fewer HEU infants mothers smoked during pregnancy (26 vs. 35%, *P* = 0.03); HEU infants had shorter breastfeeding duration, although both groups were low [1.55 (2.15) vs. 2.22 (1.88) months; *P* < 0.001] and lower SES (lowest SES 31 vs. 22%; *P* = 0.006) at 6 weeks compared with HIV-unexposed; other demographics were similar, Table 1. More HEU infants had a LRTI during the first 2 years of life than HIV-unexposed (49 vs. 37.6%, *P* = 0.04).

**Table 1:** Demographics and exposures of cohort at 6 weeks.

	Total, *n* = 909	HIV exposed, *n* = 190	HIV unexposed, *n* = 719	*P* value^a^
Sociodemographic characteristics				
Male sex	473 (52.0%)	97 (51.1%)	376 (52.3%)	0.093
Ethnicity				
African-ancestry	477 (52.5%)	177 (93.2%)	300 (41.7%)	<0.001
Mixed-ancestry	432 (47.5%)	13 (6.8%)	419 (58.3%)	–
SES quartiles				
Lowest SES	218 (24.0%)	59 (31.1%)	159 (22.1%)	0.006
Low–mod SES	237 (26.1%)	56 (29.5%)	181 (25.2%)	–
Mod–high SES	234 (25.7%)	43 (22.6%)	191 (26.6%)	–
High SES	220 (24.2%)	32 (16.8%)	188 (26.2%)	–
Preterm (<37 weeks)	140 (15.4%)	30 (15.8%)	110 (15.35)	0.879
BMI *z*-score	0.15 (1.17)	0.24 (1.21)	0.12 (1.16)	0.096
Maternal cotinine results^b^				
Nonsmoker	202 (22.8%)	42 (22.5%)	160 (22.9%)	0.026
Passive smoker	391 (44.1%)	97 (51.9%)	294 (42.0%)	–
Active smoker	294 (33.1%)	48 (25.6%)	246 (35.1%)	–
Infant cotinine results^b^				
No smoke exposure	183 (21.9%)	57 (32.2%)	126 (19.1%)	<0.001
Moderate smoke exposure	585 (70.0%)	113 (63.8%)	472 (71.6%)	–
High smoke exposure	68 (8.1%)	7 (4.0%)	61 (9.3%)	–
Duration of exclusive breastfeeding (months)	2.08 (1.96)	1.55 (2.15)	2.22 (1.88)	<0.001
LRTI during 2 years	363 (39.9%)	93 (49.0%)	270 (37.6%)	0.004
Maternal TB	41 (4.5%)	24 (12.6%)	17 (2.4%)	<0.001
Maternal HIV variables				
Maternal CD4^+^ cell count^b^				
≥500 cells/μl	72 (8.0%)	72 (40.9%)		
350–500 cells/μl	39 (4.4%)	39 (22.2%)		
<350 cells/μl	65 (7.3%)	65 (36.9%)		
Maternal VL in pregnancy^b^				
Lower than detectable limit (<40 copies/ml)	69 (8.3%)	69 (61.1%)		
VL detectable (≥40–1000 copies/ml)	26 (3.1%)	26 (23.0)		
Virally unsuppressed (>1000 copies/ml)	18 (2.2%)	18 (15.9%)		
ART regimen during pregnancy^b^				
PMTCT prophylaxis (ZDV)	29 (3.3%)	29 (15.5%)		
ART^c^	158 (17.4%)	158 (84.5%)		
First/second/third-line ART				
First-line ART	147 (16.2%)	147 (93.0%)		
Second/third-line ART	11 (0.1%)	11 (7.0%)		
ART initiation timepoint limited to current WHO first-line ART^d^				
Before pregnancy	48 (5.3%)	48/123 (39.0%)		
During pregnancy	75 (8.5%)	75/123 (61.0%)		
Infant prophylaxis				
NVP alone	165 (18.2%)	165 (87.8%)		
NVP + ZDV	23 (2.5%)	23 (12.2%)		

ART, antiretroviral treatment; D4T, stavudine; EFV, efavirenz; KAL, kaletra (lopinavir/ritonavir); LRTI, lower respiratory tract infection; NVP, nevirapine; PMTCT, prevention of mother-to-child transmission; SES, socioeconomic status; TB, tuberculosis; TDF, tenofovir; VL, viral load; ZDV, zidovudine.

^a^Unpaired *t* test used for continuous variables (means and SD presented); Chi-squared for categorical variables (*n* and % proportions presented).

^b^Missing data: maternal cotinine results had 22 missing values – three HIV exposed, and 19 HIV unexposed; infant cotinine results had 73 missing values – 13 HIV exposed and 60 HIV unexposed; maternal HIV diagnosis time point had two missing values; maternal CD4^+^ cell count had 14 missing values; maternal viral load in pregnancy had 77 missing values; ART regimen had 25 missing values; first/second/third-line therapy had three missing values; infant prophylaxis had two missing values.

^c^ART = 3TC/EFV/TDF (*n* = 123); 3TC/ZDV/EFV (*n* = 3); 3TC/ZDV/KAL (*n* = 5); 3TC/ZDV/NVP (*n* = 6); 3TC/ZDV/RIT/ATV (*n* = 1); 3TC/KAL/TDF (*n* = 5); 3TC/NVP/TDF (*n* = 11); 3TC/D4T/EFV (*n* = 2); 3TC/D4T/NVP (*n* = 2).

^d^Restricted to WHO recommended first-line ART the majority of mothers received either separately or as fixed dose combination: 3TC/EFV/TDF.

The majority of mothers, 158 (85%), were on triple therapy ART at the time of delivery [147 (93%) on first-line therapy, of which 123 (78%) were on 3TC/EFV/TDF, WHO recommended first-line therapy]. Of these 75/123 (61%) were started on therapy during pregnancy, Table 1. Twenty nine (18%) mothers were on ZDV only as per the PMTCT guidelines at the time. Seventy-two (41%) of mothers had CD4^+^ cell counts at least 500 cells/μl, 65 (37%) had low CD4^+^ cell counts (<350 cells/μl) and the rest between 350 and 500 cells/μl. Of those with viral load taken during pregnancy, the majority of mothers (69, 61%) had a suppressed HIV viral load; 18 (16%) mothers had high viral loads of more than 1000 copies/ml.

### Effects of HIV exposure on lung function

At 6 weeks of age HEU had a larger tidal volume compared with HIV-unexposed, mean (SD) 35.9 (6.3) vs. 34.6 ml (6.4); present after adjusting for confounding factors, (1.13 ml, CI: 0.02–2.23, *P* = 0.045), Table 2 and S1. This effect did not sustain over 2 years, with similar tidal volumes between groups over 2 years, Table 3.

**Table 2:** Impact of HIV, maternal disease and antiretroviral on lung function at 6 weeks.

	Log (FRC)		LCI		Log (*t*_PTEF_/*t*_E_)		Tidal volume		Log (respiratory rate)	
	Unadjusted coefficient (95% CI), *P*	Adjusted coefficient (95% CI)^a^, *P*	Unadjusted coefficient (95% CI), *P*	Adjusted coefficient (95% CI)^a^, *P*	Unadjusted coefficient (95% CI), *P*	Adjusted coefficient (95% CI)^a^, *P*	Unadjusted coefficient (95% CI), *P*	Adjusted coefficient (95% CI)^a^, *P*	Unadjusted coefficient (95% CI), *P*	Adjusted coefficient (95% CI)^a^, *P*
Maternal HIVNo	*Reference*	*Reference*(*N* = 788)	*Reference*	*Reference*(*N* = 788)	*Reference*	*Reference*(*N* = 836)	*Reference*	*Reference*(*N* = 836)	*Reference*	*Reference*(*N* = 836)
Yes	0.02 (−0.02; 0.05), 0.330	0.02 (−0.02; 0.05), 0.422	−0.01 (−0.08; 0.07), 0.818	−0.03 (−0.11; 0.06), 0.495	0.04 (−0.01; 0.10), 0.144	−0.01 (−0.07; 0.05), 0.757	**1.29 (0.23; 2.35), 0.017**	**1.13 (0.02; 2.23), 0.045**	0.02 (−0.01; 0.06), 0218	−0.01 (−0.05; 0.04), 0.802
CD4^+^ cell count (cells/μl)>500	*Reference*	*Reference* (*N* = 151)	*Reference*	*Reference* (*N* = 151)	*Reference*	*Reference* (*N* = 159)	*Reference*	*Reference* (*N* = 159)	*Reference*	*Reference* (*N* = 159)
350–500	0.00 (−0.09; 0.09), 0.991	−0.01 (−0.09; 0.08), 0.900	−0.09 (−0.26; 0.08), 0.303	−0.09 (−0.26; 0.09), 0.343	0.01 (−0.11; 0.13), 0.881	0.10 (−0.03; 0.24), 0.269	−1.91 (−4.48; 0.67), 0.145	−1.21 (−3.72; 1.30), 0.341	0.05 (−0.05; 0.15), 0.298	0.03 (−0.07; 0.13), 0.560
≤350	−0.02 (−0.10; 0.05), 0.535	−0.02 (−0.10; 0.06), 0.605	0.00 (−0.15; 0.15), 0.964	0.03 (−0.13; 0.18), 0.710	0.11 (−0.03; 0.24), 0.120	−0.01 (−0.13; 0.11), 0.891	−1.78 (−4.04; 0.48), 0.122	−1.58 (−3.78; 0.62), 0.158	0.04 (−0.04; 0.13), 0.332	0.04 (−0.05; 0.13), 0.401
CD4^+^ with unexposed groupHUU	*Reference*	*Reference* (*N* = 776)	*Reference*	*Reference* (*N* = 776)	*Reference*	*Reference* (*N* = 823)	*Reference*	*Reference* (*N* = 823)	*Reference*	*Reference* (*N* = 823)
>500	0.03 (−0.03; 0.08), 0.349	0.02 (−0.03; 0.08), 0.403	0.01 (−0.11; 0.12), 0.925	−0.02 (−0.14; 0.10), 0.724	0.02 (−0.07; 0.10), 0.71	−0.03 (−0.12; 0.06), 0.526	**2.44 (0.84; 4.03), 0.003**	2.01 (0.46; 3.56), 0.011	−0.01 (−0.07; 0.05), 0.655	−0.03 (−0.09; 0.03), 0.268
350–500	0.03 (−0.04; 0.09), 0.470	0.03 (−0.04; 0.10), 0.372	−0.08 (−0.23; 0.07), 0.274	−0.12 (−0.27; 0.04), 0.156	**0.12 (0.01; 0.23), 0.034**	0.09 (−0.03; 0.21), 0.131	0.53 (−1.59; 2.65), 0.623	0.90 (−1.17; 2.96), 0.396	0.04 (−0.04; 0.12), 0.324	−0.01 (−0.09; 0.07), 0.853
≤350	0.00 (−0.05; 0.06), 0.980	0.00 (−0.06; 0.06), 0.928	0.01 (−0.11; 0.13), 0.887	−0.00 (−0.13; 0.13), 0.988	0.03 (−0.07; 0.12), 0.594	−0.04 (−0.13; 0.06), 0.448	0.66 (−1.07; 2.39), 0.456	0.45 (−1.24; 2.14), 0.602	0.03 (−0.03; 0.09), 0.360	−0.00 (−0.07; 0.06), 0.960
Viral loadHUU	*Reference*	*Reference* (*N* = 720)	*Reference*	*Reference* (*N* = 720)	*Reference*	*Reference* (*N* = 766)	*Reference*	*Reference* (*N* = 766)	*Reference*	*Reference* (*N* = 766)
Undetectable^b^	0.01 (−0.05; 0.06), 0.791	0.00 (−0.05; 0.06), 0.938	−0.04 (−0.16; 0.08), 0.555	−0.06 (−0.19; 0.06), 0.316	0.03 (−0.06; 0.11), 0.567	−0.03 (−0.13; 0.06), 0.469	1.44 (−0.20; 3.08), 0.085	0.97 (−0.61; 2.56), 0.227	0.05 (−0.01; 0.11), 0.095	0.02 (−0.04; 0.09), 0.440
Detectable^b^	0.02 (−0.06; 0.11), 0.576	0.04 (−0.05; 0.12), 0.494	0.12 (−0.08; 0.30), 0.234	0.08 (−0.12; 0.28), 0.438	−0.09 (−0.23; 0.06), 0.231	−0.14 (−0.29; 0.01), 0.061	1.47 (−1.25; 4.18), 0.290	2.27 (−0.32; 4.86), 0.086	0.04 (−0.06; 0.13), 0.480	−0.02 (−0.12; 0.08), 0.644
Virally unsuppressed^b^	−0.02 (−0.11; 0.09), 0.773	−0.01 (−0.11; 0.09), 0.911	0.02 (−0.20; 0.25), 0.830	0.00 (−0.22; 0.23), 0.990	−0.13 (−0.29; 0.04), 0.124	**−0.17 (−0.34; −0.01), 0.044**	−0.04 (−3.12; 3.03), 0.978	0.11 (−2.74; 2.97), 0.938	−0.00 (−0.11; 0.11), 0.987	−0.02 (−0.13; 0.09), 0.666
TreatmentZDV	*Reference*	*Reference* (*N* = 160)	*Reference*	*Reference* (*N* = 160)	*Reference*	*Reference* (*N* = 169)	*Reference*	*Reference* (*N* = 169)	*Reference*	*Reference* (*N* = 169)
Triple therapy	−0.06 (−0.14; 0.03), 0.175	−0.06 (−0.15; 0.02), 0.145	0.14 (−0.04; 0.31), 0.126	0.13 (−0.05; 0.31), 0.157	**−0.21 (−0.34; −0.08), 0.002**	**−0.25 (−0.38; −0.11), <0.001**	−1.79 (−4.39; 0.80), 0.175	−1.43 (−3.99; 1.14), 0.273	0.07 (−0.03; 0.17), 0.152	0.07 (−0.03; 0.17), 0.187
Treatment with unexposed groupHUU	*Reference*	*Reference* (*N* = 788)	*Reference*	*Reference* (*N* = 788)	*Reference*	*Reference* (*N* = 836)	*Reference*	*Reference* (*N* = 836)	*Reference*	*Reference* (*N* = 836)
ZDV	0.06 (−0.02; 0.14), 0.113	0.06 (−0.02; 0.13), 0.166	−0.12 (−0.30; 0.05), 0.158	−0.14 (−0.31; 0.04), 0.127	**0.21 (0.08; 0.34), 0.001**	**0.17 (0.04; 0.30), 0.009**	**2.73 (0.28; 5.18), 0.029**	**2.29 (0.00; 4.59), 0.050**	−0.03 (−0.12; 0.06), 0.481	−0.05 (−0.14; 0.04), 0.312
Triple therapy	0.00 (−0.03; 0.04), 0.875	0.00 (−0.04; 0.04), 0.948	0.01 (−0.07; 0.09), 0.800	−0.01 (−0.10; 0.80), 0.799	0.00 (−0.06; 0.06), 0.879	−0.05 (−0.12; 0.02), 0.129	0.94 (−0.20; 2.08), 0.107	0.73 (−0.44; 1.91), 0.222	0.04 (−0.00; 0.80), 0.061	0.01 (−0.04; 0.05), 0.702

CI, confidence interval; FRC, functional residual capacity; HUU, HIV unexposed and uninfected; LCI, lung clearance index; ZDV, zidovudine. Bold text indicates P < 0.05.

^a^Multivariable models adjusted for socioeconomic status, race, sex, BMI for age, maternal smoke (urine cotinine results).

^b^Undetectable: less than 40 copies/ml; detectable: 40–1000 copies/ml; virally unsuppressed: >1000 copies/ml.

**Table 3:** Impact of HIV, maternal disease and antiretroviral on lung growth and function over 2 years.

	Log (FRC)		LCI		Log (*t*_PTEF_/*t*_E_)		Tidal volume		Log (respiratory rate)	
	Unadjusted coefficient (95% CI), *P*	Adjusted coefficient (95% CI)^a^, *P*	Unadjusted coefficient (95% CI), *P*	Adjusted coefficient (95% CI)^a^, *P*	Unadjusted coefficient (95% CI), *P*	Adjusted coefficient (95% CI)^a^, *P*	Unadjusted coefficient (95% CI), *P*	Adjusted coefficient (95% CI)^a^, *P*	Unadjusted coefficient (95% CI), *P*	Adjusted coefficient (95% CI)^a^, *P*
Maternal HIVNo	*Reference*	*Reference* (*N* = 977)	*Reference*	*Reference* (*N* = 977)	*Reference*	*Reference* (*N* = 986)	*Reference*	*Reference* (*N* = 986)	*Reference*	*Reference* (*N* = 986)
Yes	0.01 (−0.05; 0.07), 0.792	0.02 (−0.05; 0.08), 0.628	**0.06 (0.01; 0.12), 0.030**	0.03 (−0.03; 0.10), 0.313	0.03 (−0.02; 0.07), 0.235	−0.03 (−0.08; 0.02), 0.221	2.39 (−0.145; 6.24), 0.223	2.87 (−1.41; 7.15), 0.189	0.02 (−0.01; 0.05), 0.217	−0.02 (−0.06; 0.01), 0.227
CD4^+^ cell count (cells/μl)>500	*Reference*	*Reference* (*N* = 195)	*Reference*	*Reference* (*N* = 195)	*Reference*	*Reference* (*N* = 196)	*Reference*	*Reference* (*N* = 196)	*Reference*	*Reference* (*N* = 196)
350–500	−0.06 (−0.20; 0.09), 0.449	−0.01 (−0.15; 0.14), 0.902	−0.00 (−0.15; 0.14), 0.953	−0.01 (−0.15; 0.13), 0.847	0.05 (−0.06; 0.16), 0.371	0.02 (−0.09; 0.13), 0.700	−3.60 (−13.00; 5.80), 0.452	−0.79 (−10.20; 8.62), 0.869	0.06 (−0.02; 0.14), 0.171	0.04 (−0.04; 0.12), 0.325
≤350	−0.06 (−0.18; 0.07), 0.373	−0.03 (−0.15; 0.09), 0.657	0.08 (−0.04; 0.20), 0.216	0.08 (−0.03; 0.20), 0.165	−0.00 (−0.094; 0.09), 0.971	−0.04 (−0.13; 0.06), 0.422	−4.23 (−12.37; 3.92), 0.309	−2.48 (−10.52; 5.57), 0.546	0.05 (−0.02; 0.12), 0.127	0.04 (−0.03; 0.11), 0.221
CD4^+^ with unexposed groupHUU	*Reference*	*Reference* (*N* = 960)	*Reference*	*Reference* (*N* = 960)	*Reference*	*Reference* (*N* = 969)	*Reference*	*Reference* (*N* = 969)	*Reference*	*Reference* (*N* = 969)
>500	0.03 (−0.06; 0.12), 0.493	0.02 (−0.07; 0.12), 0.657	0.04 (−0.05; 0.13), 0.342	0.02 (−0.07; 0.12), 0.609	0.02 (−0.05; 0.08), 0.573	−0.02 (−0.09; 0.04), 0.479	4.23 (−1.55; 10.01), 0.151	3.87 (−2.18; 9.92), 0.210	−0.01 (−0.06; 0.04), 0.617	−0.05 (−0.10; 0.01), 0.083
350–500	−0.02 (−0.15; 0.10), 0.698	0.01 (−0.11; 0.14), 0.835	0.04 (−0.08; 0.16), 0.487	−0.02 (−0.14; 0.11), 0.804	0.07 (−0.02; 0.16), 0.132	0.01 (−0.08; 0.10), 0.833	0.63 (−7.21; 8.46), 0.875	2.77 (−5.27; 10.82), 0.499	0.04 (−0.02; 0.11), 0.212	−0.02 (−0.09; 0.05), 0.636
≤350	−0.02 (−0.12; 0.07), 0.617	−0.01 (−0.11; 0.09), 0.893	**0.11 (0.02; 0.21), 0.017**	0.08 (−0.02; 0.18), 0.129	0.02 (−0.05; 0.09), 0.635	−0.05 (−0.12; 0.02), 0.187	0.01 (−6.27; 6.28), 0.998	0.59 (−5.91; 7.09), 0.859	0.04 (−0.01; 0.10), 0.136	−0.01 (−0.06; 0.05), 0.839
Viral loadHUU	*Reference*	*Reference* (*N* = 894)	*Reference*	*Reference* (*N* = 894)	*Reference*	*Reference* (*N* = 902)	*Reference*	*Reference* (*N* = 902)	*Reference*	*Reference* (*N* = 902)
Undetectable^b^	0.05 (−0.04; 0.14), 0.252	0.04 (−0.06; 0.13), 0.463	0.01 (−0.08; 0.10), 0.847	−0.02 (−0.10; 0.09), 0.963	0.03 (−0.03; 0.09), 0.365	−0.03 (−0.10; 0.04), 0.417	4.18 (−1.55; 9.92), 0.153	3.52 (−2.54; 9.58), 0.255	0.03 (−0.02; 0.08), 0.307	−0.01 (−0.06; 0.04), 0.696
Detectable^b^	0.01 (−0.14; 0.16), 0.916	0.06 (−0.09; 0.21), 0.452	0.12 (−0.02; 0.27), 0.102	0.07 (−0.08; 0.22), 0.380	−0.05 (−0.16; 0.06), 0.379	**−0.12 (−0.23; −0.01), 0.039**	5.44 (−4.13; 15.01), 0.265	8.12 (−1.51; 17.75), 0.098	0.02 (−0.06; 0.10), 0.633	−0.04 (−0.13; 0.04), 0.308
Virally unsuppressed^b^	−0.00 (−0.17; 0.17), 0.994	0.01 (−0.15; 0.18), 0.874	**0.22 (0.06; 0.39), 0.009**	**0.17 (0.00; 0.34), 0.046**	−0.08 (−0.20; 0.04), 0.208	**−0.13 (−0.26; −0.01), 0.037**	3.18 (−7.65; 14.01), 0.565	4.22 (−6.57; 15.01), 0.444	0.01 (−0.09; 0.10), 8.94	−0.04 (−0.13; 0.06), 0.436
TreatmentZDV	*Reference*	*Reference* (*N* = 209)	*Reference*	*Reference* (*N* = 209)	*Reference*	*Reference* (*N* = 210)	*Reference*	*Reference* (*N* = 210)	*Reference*	*Reference* (*N* = 210)
Triple therapy	0.04 (−0.11; 0.19), 0.605	0.02 (−0.13; 0.16), 0.797	0.06 (−0.08; 0.20), 0.422	0.06 (−0.08; 0.20), 0.406	**−0.12 (−0.23; −0.01), 0.031**	**−0.14 (−0.25; −0.03), 0.015**	4.27 (−5.54; 14.10), 0.394	3.66 (−6.01; 13.32), 0.458	0.01 (−0.07; 0.10), 0.773	0.01 (−0.08; 0.09), 0.844
Treatment with unexposed groupHUU	*Reference*	*Reference* (*N* = 977)	*Reference*	*Reference* (*N* = 977)	*Reference*	*Reference* (*N* = 986)	*Reference*	*Reference* (*N* = 986)	*Reference*	*Reference* (*N* = 986)
ZDV	−0.02 (−0.16; 0.12), 0.759	−0.01 (−0.15; 0.13), 0.888	0.02 (−0.12; 0.16), 0.828	−0.02 (−0.16; 0.12), 0.751	**0.13 (0.02; 0.23), 0.019**	0.08 (−0.02; 0.19), 0.128	−1.07 (−10.35; 8.21), 0.821	−0.61 (−9.83; 8.60), 0.896	0.01 (−0.07; 0.09), 0.810	−0.02 (−0.10; 0.06), 0.557
Triple therapy	0.02 (−0.05; 0.08), 0.608	0.02 (−0.05; 0.10), 0.497	**0.07 (0.01; 0.13), 0.033**	0.04 (−0.03; 0.11), 0.288	0.00 (−0.04; 0.05), 0.888	**−0.06 (−0.11; −0.01), 0.028**	3.20 (−0.91; 7.31), 0.127	3.68 (−0.86; 0.821), 0.112	0.02 (−0.01; 0.06), 0.219	−0.02 (−0.06; 0.02), 0.256

CI, confidence interval; FRC, functional residual capacity; HUU, HIV unexposed and uninfected; LCI, lung clearance index; ZDV, zidovudine. Bold text indicates P < 0.05.

^a^Multivariable mixed effect models adjusted for socioeconomic status, race, sex, BMI for age, maternal smoke (cotinine results), LRTI.

^b^Undetectable: less than 40 copies/ml; detectable: 40–1000 copies/ml; virally unsuppressed: more than 1000 copies/ml.

The LCI was higher in HEU compared with HIV-unexposed at both 12 (6.8 vs. 6.7, *P* = 0.032) and 24 months (6.8 vs. 6.7, *P* = 0.003); this was an average 0.12 (CI 0.02–0.23, *P* = 0.020) turnovers higher at 2 years compared with HIV-unexposed, Table S1. However, this trend was not statistically significant over 2 years in the multivariate model, Table 3. HEU had a higher average respiratory rate compared with HIV-unexposed at 12 months (30.6 vs. 29.5, *P* = 0.007) which was not seen at 24 months, Table S1. The FRC and *t*_PTEF_/*t*_E_ were similar between groups.

### Effects of maternal HIV disease and antiretroviral exposure on lung function

#### Impact of maternal CD4^+^ cell count

Compared with HIV-unexposed, infants whose mothers had a CD4^+^ cell count more than 500 cells/μl had higher tidal volumes at 6 weeks, average 2.0 ml (CI 0.46–3.56, *P* = 0.01), Table 2 compared with those less than 500 cells/μl. Over 2 years, a low maternal CD4^+^ cell count (<350 cells/μl) was associated with a higher LCI (0.1, CI 0.02–0.21, *P* = 0.02), however, this was NS when adjusted for potential confounders. Maternal CD4^+^ cell count during pregnancy had no effect on FRC, respiratory rate nor *t*_PTEF_/*t*_E_ measures at 2 years. However, in those children exposed to first-line triple ART, low maternal CD4^+^ cell counts were associated with lower infant tidal volume over 2 years, average 11.1 ml lower (CI −18.58–3.58, *P* = 0.004), Table 4.

**Table 4:** Antiretroviral therapy started before and during pregnancy (limited to children exposed to triple therapy currently recommended by WHO) with maternal CD4^+^ cell count at 6 weeks and 24 months.

	Log (FRC)		LCI		Log (*t*_PTEF_/*t*_E_)		Tidal volume		Log (respiratory rate)	
	6 Weeks: adjusted coefficient (95% CI)^a^, *P*	24 Months: adjusted coefficient (95% CI)^b^, *P*	6 Weeks: adjusted coefficient (95% CI)^a^, *P*	24 Months: adjusted coefficient (95% CI)^b^, *P*	6 Weeks: adjusted coefficient (95% CI)^a^, *P*	24 Months: adjusted coefficient (95% CI)^b^, *P*	6 Weeks: adjusted coefficient (95% CI)^a^, *P*	24 Months: adjusted coefficient (95% CI)^b^, *P*	6 Weeks: adjusted coefficient (95% CI)^a^, *P*	24 Months: adjusted coefficient (95% CI)^b^, *P*
ART initiationBefore pregnancy	*Reference* (*N* = 95)	*Reference* (*N* = 72)	*Reference* (*N* = 95)	*Reference* (*N* = 72)	*Reference* (*N* = 100)	*Reference* (*N* = 76)	*Reference* (*N* = 100)	*Reference* (*N* = 76)	*Reference* (*N* = 100)	*Reference* (*N* = 76)
During pregnancy	−0.08 (−0.18; 0.01),0.073	0.02 (−0.07; 0.11),0.655	−0.12 (−0.31; 0.08),0.237	−0.04 (−0.31; 0.22),0.743	0.06 (−0.08; 0.20),0.390	0.17 (−0.01; 0.35),0.059	**−2.70 (−5.31; −0.10),0.042**	−5.67 (−12.82; 1.48),0.118	0.06 (−0.05; 0.17),0.275	0.05 (−0.01; 0.12),0.120
CD4^+^ cell count (cells/μl)>500	*Reference*	*Reference*	*Reference*	*Reference*	*Reference*	*Reference*	*Reference*	*Reference*	*Reference*	*Reference*
350–500	0.03 (−0.09; 0.15),0.612	−0.06 (−0.19; 0.06),0.302	−0.23 (−0.48; 0.03),0.085	−0.11 (−0.46; 0.23),0.513	0.16 (−0.03; 0.35),0.091	−0.04 (−0.29; 0.20),0.726	−2.19 (−5.61; 1.24),0.208	**−11.70 (−21.46; −1.94),0.020**	0.03 (−0.11; 0.17),0.705	0.00 (−0.09; 0.09),0.969
≤350	0.05 (−0.06; 0.15),0.367	−0.03 (−0.13; 0.06),0.489	−0.09 (−0.31; 0.12),0.390	−0.10 (−0.37; 0.17),0.469	0.14 (−0.02; 0.29),0.091	0.03 (−0.16; 0.22),0.749	−1.42 (−4.30; 1.46),0.329	**−11.08 (−18.58; −3.58),0.004**	0.02 (−0.10; 0.14),0.763	0.06 (−0.01; 0.13),0.087

ART, antiretroviral therapy; CI, confidence interval; FRC, functional residual capacity; LCI, lung clearance index. Bold text indicates P < 0.05.

^a^6-Week multivariable models include ART initiation, CD4^+^ categorical variables, socioeconomic status, race, sex, BMI for age, maternal smoke (cotinine results); only HIV variables (ART and CD4^+^ shown).

^b^24-Month multivariable models include ART initiation, CD4^+^ categorical variables, socioeconomic status, race, sex, BMI for age, infant smoke exposure (infant cotinine results), LRTI; only HIV variables (ART and CD4^+^ shown).

#### Impact of maternal HIV viral load

Having a mother with an unsuppressed HIV viral load (>1000 copies/ml), was associated with a 17% lower expiratory flow ratio time to peak tidal expiratory flow over total expiratory time (*t*_PTEF_/*t*_E_) at 6 weeks, CI −0.34 to −0.01, *P* = 0.044, Table 2. Over 2 years, a high maternal viral load (>1000 copies/ml) was associated with an increased LCI (0.17, CI 0.00–0.34, *P* = 0.046) and a 13% lower *t*_PTEF_/*t*_E_ (CI −0.26 to −0.01, *P* = 0.037), Table 3. Maternal viral load was not associated with respiratory rate, tidal volume nor FRC over 2 years.

#### The impact of antiretroviral exposure on lung function

Compared with HIV-unexposed infants, infants exposed to ZDV had a higher *t*_PTEF_/*t*_E_ (0.17, 0.04–0.30, *P* = 0.009) and higher tidal volumes (2.37, CI 0.08–4.67, 0.043) at 6 weeks, Table 2. Among the HEU, those exposed to triple ART, had lower *t*_PTEF_/*t*_E_ at 6 weeks compared with infants exposed to ZDV (−0.30, −0.45–0.14, *P* < 0.001), an effect that was not sustained over 2 years, Table S2. In analyses limited to infants exposed to current WHO first-line triple ART (Table 4), ART initiated during pregnancy was associated with lower infant tidal volumes at 6 weeks compared with those who had ART initiated prior to pregnancy, average 2.7 ml lower (−5.31 to −0.10, *P* = 0.042).

## Discussion

The current article provides novel data on the impact of HIV and antiretroviral exposure on the developing lung function trajectory over the first 2 years of life in an African birth cohort from a high HIV prevalence area. We show that HIV exposure is associated with altered lung function soon after birth and at 2 years of age. Overall these effects were mild. The study identified both uncontrolled maternal HIV disease (as measured by high HIV viral load), immunological compromise and ART initiation during pregnancy as factors associated with poorer lung function outcomes.

The impact of HIV exposure on tidal volume at 6 weeks has been previously published [17]. The 6-week lung function reflects in part the impact of the antenatal environment when the foetus is more directly exposed to their mother's immunity, antiretrovirals and potentially the HIV virus. The average increase in tidal volume was small, but statistically significant even after correcting for somatic growth and other predictors. We had previously hypothesised that the increased tidal volume could be related to breathing control affected by antiretrovirals, although respiratory rate and expiratory flow ratios were not different. ZDV exposure in children can cause anaemia and lactic acidosis, both may affect breathing pattern [26]. However, we did not have data to assess this in our cohort; in addition the numbers of children exposed to ZDV was small and should be interpreted with caution. This association warrants further investigation, although with current PMTCT recommendations HEU infants are unlikely to be exposed to ZDV antenatally in the future; however, they continue to have postnatal exposure prophylaxis [20].

The early effects of HIV on tidal volume were not sustained over 2 years and, in fact, HIV exposure had no independent effect on any lung function outcomes over 2 years. This is interesting in the context of current literature emerging around HEU children. A recent study found an association between HIV exposure without infection and early child growth trajectories [27]. In addition, another study from the DCHS found HEU children were at increased risk of hospitalized LRTI in the first 6 months of life [12]. It is likely all of these variables are interlinked, and we corrected for LRTI and child growth in the multivariate models. Although we did not find an effect of maternal HIV alone, we found associations with maternal viral load and CD4^+^ cell count.

In this study, HEU infants whose mothers had high HIV viral loads, had higher LCI at 2 years, evidence of the association between maternal disease status and infant respiratory health. A high maternal viral load was also associated with reduced expiratory flow ratios (*t*_PTEF_/*t*_E_) at 6 weeks, which persisted over 2 years. This effect was present even after correcting for early life LRTI and other predictors. These data suggest that a subgroup of HEU children whose mothers have more advanced HIV disease may be most vulnerable to lung function impairment and require closer follow-up.

Assessing the impact of antiretrovirals on lung function was affected by our cohort being a heterogeneous group, which is similar to other SSA settings. The PMTCT guidelines, as well as adult ART initiation guidelines, changed during the early stages of the cohort follow-up and hence not all infants were exposed to the same maternal ART regimens; however, the majority of mothers were on WHO recommended first-line triple therapy reflecting current PMTCT guidelines. When analysing this group separately, initiation of ART during pregnancy and low CD4^+^ cell counts were associated with lower tidal volume at 6 weeks and over 2 years, respectively. This suggests that earlier ART initiation in mothers is beneficial to infant lung growth, highlighting the importance of maternal health in influencing child respiratory outcomes. This is similar to a recent study from Belgium which showed that maternal ART initiation before pregnancy reduced the infection-related hospitalization risk and had associations with the HEU infant immunological status [16].

The current study has many strengths. It is a large birth cohort within a high HIV prevalence area, followed longitudinally through to 2 years with good retention of 86%. Comprehensive lung function measures were collected at birth and annually, allowing the assessment of exposure impact over time. There was robust collection of other risk factor information such as tobacco smoke exposure and early life LRTI, allowing us to account for these factors in analysis. The fact that ART treatment protocols were not consistent during the early study, meant that there was heterogeneity of ART exposure and maternal disease markers which is reflective of many other SSA countries and hence speaks to the generalizability of this data. A limitation of the study is that we had a large amount of missing viral load data, which reduced our statistical power to assess the impact of HIV viral load on outcomes and further work is needed to replicate our findings. In addition potential confounders excluded may have an impact on our analysis, but we feel that the covariates included reduce residual confounding.

### Conclusion

This is the first study to comprehensively assess the impact of HIV exposure and ART on infant lung function and growth during early life. It shows that HIV exposure has mild impacts on lung function and growth during early life and that maternal disease severity as measured by low CD4^+^ cell count and high HIV viral load increases risk of lung function impairment. These findings need to be validated in other cohort studies, where HIV and respiratory disease risk factor prevalences differ. The impact of antiretrovirals and timing of ART initiation support the current WHO guidelines; however, the longer term implications of these findings require further investigation, which we are undertaking. This study highlights the importance of monitoring and follow-up of HEU infants, particularly those whose mothers have poor disease control.

## Acknowledgements

We thank the mothers and children for participating in the study and the study staff, the clinical and administartiove staff from the Western Cape Department of Health at Paarl Hospital and the clinics for the support of the study. The study is funded by the Wellcome Trust (#204755/Z/16/Z, #098479/Z/12/Z, #203525/Z/16/Z), Bill and Melinda Gates Foundation (OPP1017641), Worldwide University Network Research Mobility Award, University of Cape Town equipment grant and Thrasher Foundation (#9207).

### Conflicts of interest

There are no conflicts of interest.

## Supplementary Material

Supplemental Digital Content
