# Supplementary material for: Impact of HIV and antiretroviral drug exposure on lung growth and function over 2 years in an African Birth Cohort
Source: AIDS. 2019 Nov 11;34(4):549–58. doi: 10.1097/QAD.0000000000002444 (PMC7050792; doi:10.1097/QAD.0000000000002444)
Supplement: Supplemental Digital Content [file aids-34-549-s001.doc]

**Supplementary material: Impact of HIV and antiretroviral drug exposure on lung growth and function over 2 years in an African Birth Cohort**

DM Gray^1^, CJ Wedderburn^1,2^, RP MacGinty^1^, L McMillan^1^, C Jacobs^1^, JA Stadler^1^, GL Hall^3^, HJ Zar^1^

^1^Department of Paediatrics and Child Health, Red Cross War Memorial Children’s Hospital and MRC Unit on Child and Adolescent Health, University of Cape Town, Cape Town, South Africa;^2^Department of Clinical Research, London School of Hygiene & Tropical Medicine, London, UK; ^3^Telethon Kids Institute, School of Physiotherapy and Exercise Science, Curtin University, and Centre for Child Health Research, University of Western Australia, Perth, Australia

HIV exposure

Supplementary table S1: Comparison between those attending and not attending (including lost to follow up) at 12-month visit

|  | **Total^1^**  **(n=1141)** | **Attended**  **(n=782)** | **Did not attend (n=359)** | **P-value^2^** |
| --- | --- | --- | --- | --- |
| ***Socio-demographic characteristics*** | | | | |
| Male sex | 587 (51.5%) | 398 (50.9%) | 189 (52.7%) | 0.583 |
| Ethnicity |  |  |  |  |
| African-ancestry | 630 (55.2%) | 394 (50.4%) | 236 (65.7%) | <0.001 |
| Mixed-ancestry | 511 (44.8%) | 388 (49.6%) | 123 (34.3%) | - |
| SES Quartiles |  |  |  |  |
| Lowest SES | 273 (23.9%) | 184 (23.5%) | 89 (24.8%) | 0.156 |
| Low-Mod SES | 296 (25.9%) | 210 (26.9%) | 86 (24.0%) | - |
| Mod-High SES | 290 (25.4%) | 208 (26.6%) | 82 (22.8%) | - |
| High SES | 282 (24.7%) | 180 (23.0%) | 102 (28.4%) | - |
| Preterm (< 37 weeks) | 194 (17.0%) | 122 (15.6%) | 72 (20.1%) | 0.063 |
| HIV exposure | 246 (21.6%) | 172 (22.0%) | 74 (20.7%) | 0.622 |
| CD4 (cells/mm3): |  |  |  |  |
| >500 | 87/207 (42.0%) | 65/157 (41.4%) | 22/50 (44.0%) | 0.926 |
| 350 -500 | 45/ 207 (21.7%) | 34/157 (21.7%) | 11/50 (22.0%) | - |
| < 350 | 75/207 (36.2%) | 58/157 (36.9%) | 17/50 (34.0%) | - |
| CD4 with unexposed group |  |  |  |  |
| HUU | 808/1015 (79.6%) | 610/767 (79.5%) | 198/248 (79.8%) | 0.983 |
| >500 | 87/1015 (8.6%) | 65/767 (8.5%) | 22/248 (8.9%) | - |
| 350 -500 | 45/1015 (4.4%) | 34/767 (4.4%) | 11/248 (4.4%) | - |
| < 350 | 75/1015 (7.4%) | 58/767 (7.6%) | 17/248 (6.9%) | - |
| Viral Load^3^ |  |  |  |  |
| HUU | 808/944 (85.6%) | 610/717 (85.1%) | 198/227 (87.2%) | 0.815 |
| Undetectable | 88/944 (9.3%) | 68/717 (9.5%) | 20/227 (8.8%) | - |
| Detectable | 28/944 (3.0%) | 23/717 (3.2%) | 5/227 (2.2%) | - |
| Virally unsuppressed | 20/944 (2.1%) | 16/717 (2.2%) | 4/227 (1.8%) |  |
| Weight for age Z-score at birth | -0.55 (1.14) | -0.55 (1.17) | -0.56 (1.06) | 0.632 |
| Maternal cotinine results |  |  |  |  |
| Non-smoker | 261/1091 (23.9%) | 165 (21.9%) | 96 (28.6%) | 0.009 |
| Passive smoker | 479/1091 (43.9%) | 328 (43.4%) | 151 (44.9%) | - |
| Active smoker | 351/1091 (32.2%) | 262 (34.7%) | 89 (26.5%) | - |
| Infant cotinine results |  |  |  |  |
| No smoke exposure | 311/1007 (30.9%) | 198 (26.4%) | 113 (44.0%) | <0.001 |
| Moderate smoke exposure | 578/1007 (57.4%) | 457 (60.9%) | 121 (47.0%) | - |
| High smoke exposure | 118/1007 (11.7%) | 95 (12.7%) | 23 (9.0%) | - |
| Duration of exclusive breastfeeding (months) | 1.94/1007 (1.94) | 2.01 (1.99) | 1.73 (1.80) | 0.077 |
| Maternal TB | 54 (4.7%) | 40 (5.1%) | 14 (3.9%) | 0.369 |

HUU= HIV uninfected and unexposed

^1^ 2 children excluded as HIV positive

^2^ Unpaired t-test used for continuous variables (means and SD presented); Chi-squared for categorical variables (n and % proportions presented).

^3^ Undetectable: <40 copies/mL; detectable: 40-1000 copies/mL; virally unsuppressed: >1000 copies/mL

Notes – could remove maternal cotinine here – used infant smoke in model.

Differences in races between those attending and not attending - higher percentage of African-ancestry in those not attending

Differences in smoke between those attending and those not attending – higher percentage of children with high exposure compared to those not attending

Supplementary table S2: Comparison between those attending and not attending (including lost to follow up) at 24-month visit

|  | **Total^1^**  **(n=1141)** | **Attended**  **(n=740)** | **Did not attend (n=401)** | **P-value^2^** |
| --- | --- | --- | --- | --- |
| ***Socio-demographic characteristics*** | | | | |
| Male sex | 587 (51.5%) | 382 (51.6%) | 205 (51.1%) | 0.872 |
| Ethnicity |  |  |  |  |
| African-ancestry | 630 (55.2%) | 363 (49.1%) | 267 (66.6%) | <0.001 |
| Mixed-ancestry | 511 (44.8%) | 377 (51.0%) | 134 (33.4%) | - |
| SES Quartiles |  |  |  |  |
| Lowest SES | 273 (23.9%) | 168 (22.7%) | 105 (26.2%) | 0.092 |
| Low-Mod SES | 296 (25.9%) | 196 (26.5%) | 100 (24.9%) | - |
| Mod-High SES | 290 (25.4%) | 203 (27.4%) | 87 (21.7%) | - |
| High SES | 282 (24.7%) | 173 (23.4%) | 109 (27.2%) | - |
| Preterm (< 37 weeks) | 194 (17.0%) | 117 (15.8%) | 77 (19.2%) | 0.145 |
| HIV exposure | 246 (21.6%) | 154 (20.8%) | 92 (23.1%) | 0.386 |
| CD4 (cells/mm3): |  |  |  |  |
| >500 | 87/207 (42.0%) | 61/140 (43.6%) | 26/67 (38.8%) | 0.694 |
| 350 -500 | 45/ 207 (21.7%) | 31/140 (22/1%) | 14/67 (20.9%) | - |
| < 350 | 75/207 (36.2%) | 48/140 (34.3%) | 27/67 (40.3%) | - |
| CD4 with unexposed group |  |  |  |  |
| HUU | 808/1015 (79.6%) | 586/726 (80.7%) | 222/289 (76.8%) | 0.437 |
| >500 | 87/1015 (8.6%) | 61/726 (8.4%) | 26/289 (9.0%) | - |
| 350 -500 | 45/1015 (4.4%) | 31/726 (4.3%) | 14/289 (4.8%) | - |
| < 350 | 75/1015 (7.4%) | 48/726 (6.6%) | 27/289 (9.4%) | - |
| Viral Load^3^ |  |  |  |  |
| HUU | 808/944 (85.6%) | 586/689 (85.1%) | 222/255 (87.1%) | 0.396 |
| Undetectable | 88/944 (9.3%) | 63/689 (9.1%) | 25/255 (9.8%) | - |
| Detectable | 28/944 (3.0%) | 24/689 (3.5%) | 4/255 (1.6%) | - |
| Virally unsuppressed | 20/944 (2.1%) | 16/689 (2.3%) | 4/255 (1.6%) | - |
| Weight for age Z-score at birth | -0.55 (1.14) | -0.58 (1.17) | -0.49 (1.06) | 0.215 |
| Maternal cotinine results^*^ |  |  |  |  |
| Non-smoker | 261 (23.9%) | 154 (21.5%) | 107 (28.7%) | 0.003 |
| Passive smoker | 479 (43.9%) | 311 (43.3%) | 168 (45.0%) | - |
| Active smoker | 351 (32.2%) | 253 (35.2%) | 98 (26.3%) | - |
| Infant cotinine results^*^ |  |  |  | - |
| No smoke exposure | 311 (30.9%) | 193 (26.8%) | 118 (41.3%) | <0.001 |
| Moderate smoke exposure | 578 (57.4%) | 438 (60.8%) | 140 (49.0%) | - |
| High smoke exposure | 118 (11.7%) | 90 (12.4%) | 28 (9.7%) | - |
| Duration of exclusive breastfeeding (months) | 1.94 (1.94) | 2.05 (1.95) | 1.68 (1.92) | <0.001 |
| Maternal TB | 58 (5.1%) | 42 (5.7%) | 16 (4.0%) | 0.216 |

HUU= HIV uninfected and unexposed

^1^ 2 children excluded as HIV positive

^2^ Unpaired t-test used for continuous variables (means and SD presented); Chi-squared for categorical variables (n and % proportions presented).

^3^ Undetectable: <40 copies/mL; detectable: 40-1000 copies/mL; virally unsuppressed: >1000 copies/mL

Notes – could remove maternal cotinine here – used infant smoke in model.

Differences in races between those attending and not attending - higher percentage of African-ancestry in those not attending

Differences in smoke between those attending and those not attending – higher percentage of children with high exposure compared to those not attending

Differences in duration of breast feeding – higher duration of breastfeeding in those attending visit vs those not attending

Table S3 Lung function outcomes at 6 weeks, 1 year and 2 years by HIV exposure

|  | **Total**  **Mean (SD)** | **HIV exposed**  **Mean (SD)** | **HIV unexposed**  **Mean (SD)** | **P-value** |
| --- | --- | --- | --- | --- |
| ***Lung function tests at 6 weeks*** | | | | |
| **Multiple breath washout measures (n= 809; *166 HIV exposed; 643 HIV unexposed*)** | | | | |
| FRC (L) | 0.08 (0.02) | 0.08 (0.02) | 0.08 (0.02) | 0.283 |
| LCI | 7.16 (0.44) | 7.15 (0.41) | 7.16 (0.45) | 0.589 |
| **Tidal breathing measures (n= 857; *175 HIV exposed; 682 HIV unexposed*)** | | | | |
| TPTEF/tE (%) | 38.30 (12.23) | 39.46 (12.28) | 38.01 (12.21) | 0.167 |
| Tidal volume (mL) | 34.83 (6.38) | 35.86 (6.25) | 34.57 (6.39) | 0.019 |
| Respiratory rate (min) | 48.71 (11.37) | 49.70 (11.55) | 48.46 (11.31) | 0.131 |
| ***Lung function tests at 12 months*** | | | | |
| **Multiple breath washout measures (n= 688; *147 HIV exposed; 541 HIV unexposed*)** | | | | |
| FRC (L) | 0.20 (0.04) | 0.20 (0.04) | 0.20 (0.04) | 0.808 |
| LCI (%) | 6.74 (0.61) | 6.82 (0.54) | 6.72 (0.63) | 0.032 |
| **Tidal breathing measures (n= 729; *156 HIV exposed; 573 HIV unexposed*)** | | | | |
| TPTEF/tE | 30.01 (10.74) | 31.02 (10.93) | 29.73 (10.68) | 0.168 |
| Tidal volume (mL) | 91.92 (14.27) | 93.19 (14.23) | 91.57 (14.27) | 0.338 |
| Respiratory rate (min) | 29.72 (5.03) | 30.61 (5.09) | 29.47 (4.99) | 0.007 |
| ***Lung function tests at 24 months*** | | | | |
| **Multiple breath washout measures (n= 621; *127 HIV exposed; 494 HIV unexposed*)** | | | | |
| FRC (L) | 0.26 (0.05) | 0.26 (0.05) | 0.26 (0.05) | 0.184 |
| LCI | 6.68 (0.47) | 6.78 (0.45) | 6.65 (0.47) | 0.003 |
| **Tidal breathing measures (n= 646; *137 HIV exposed; 509 HIV unexposed*)** | | | | |
| TPTEF/tE (%) | 27.69 (9.80) | 27.90 (10.28) | 27.63 (9.67) | 0.962 |
| Tidal volume (mL) | 118.93 (16.78( | 120.37 (15.96) | 118.55 (16.99) | 0.353 |
| Respiratory rate (min) | 26.41 (4.41) | 26.75 (3.84) | 26.32 (4.55) | 0.141 |

FRC = Functional residual capacity; LCI = Lung clearance index; TPTEF/tE= Ratio time of Peak Total Expiratory Flow to time of Expiration

Table S2 ART (AZT versus triple therapy) with maternal CD4 included at 6 weeks and 2 years

|  | **Log (FRC)** | | **LCI** | | **Log (tptef/te)** | | **Tidal Volume** | | **Log (Respiratory rate)** | |
| --- | --- | --- | --- | --- | --- | --- | --- | --- | --- | --- |
|  | **6 weeks:**  **Adjusted**  **coefficient**  **(95% CI)^*^, p** | **24 months:**  **Adjusted coefficient**  **(95% CI)^**^, p** | **6 weeks:**  **Adjusted**  **coefficient**  **(95% CI)^*^, p** | **24 months:**  **Adjusted coefficient**  **(95% CI)^**^, p** | **6 weeks:**  **Adjusted**  **coefficient**  **(95% CI)^*^, p** | **24 months:**  **Adjusted coefficient**  **(95% CI)^**^, p** | **6 weeks:**  **Adjusted**  **coefficient**  **(95% CI)^*^, p** | **24 months:**  **Adjusted coefficient**  **(95% CI)^**^, p** | **6 weeks:**  **Adjusted**  **coefficient**  **(95% CI)^*^, p** | **24 months:**  **Adjusted coefficient**  **(95% CI)^**^, p** |
| Treatment:  AZT | *Reference*  *(N=121)* | *Reference*  *(N=88)* | *Reference*  *(N=121)* | *Reference*  *(N=88)* | *Reference*  *(N=127)* | *Reference*  *(N=93)* | *Reference*  *(N=127)* | *Reference*  *(N=93)* | *Reference*  *(N=127)* | *Reference*  *(N=93)* |
| Triple therapy | -0.09 (-0.19; 0.02), 0.100 | -0.04 (-0.15; 0.07), 0.429 | 0.15 (-0.06; 0.36), 0.148 | 0.05 (-0.23; 0.33), 0.731 | **-0.30 (-0.45; -0.14), <0.001** | 0.01 (-0.21; 0.22), 0.956 | -1.04 (-4.04; 1.96), 0.494 | 0.26 (-8.28; 8.81), 0.951 | 0.08 (-0.45; 0.20), 0.186 | 0.02 (-0.07; 0.10), 0.699 |
| CD4 (cells/mm3):  > 500 | *Reference* | *Reference* | *Reference* | *Reference* | *Reference* | *Reference* | *Reference* | *Reference* | *Reference* | *Reference* |
| 350-500 | 0.03 (-0.07; 0.12), 0.604 | -0.06 (-0.16; 0.05), 0.279 | -0.10 (-0.30; 0.10), 0.308 | 0.01 (-0.27; 0.27), 0.992 | 0.14 (-0.00; 0.29), 0.056 | -0.03 (-0.23; 0.17), 0.769 | -1.83 (-4.68; 1.02), 0.207 | -4.53 (-12.67; 3.61), 0.272 | 0.02 (-0.09; 0.14), 0.693 | 0.02 (-0.06; 0.09), 0.670 |
| <=350 | 0.03 (-0.06; 0.12), 0.562 | -0.03 (-0.12; 0.06), 0.525 | -0.06 (-0.25; 0.13), 0.536 | -0.02 (-0.27; 0.22), 0.848 | 0.12 (-0.02; 0.26), 0.082 | 0.08 (-0.11; 0.26), 0.412 | -1.60 (-4.35; 1.14), 0.250 | **-9.43 (-16.67; -2.18), 0.011** | 0.01 (-0.10; 0.12), 0.825 | **0.08 (0.10; 0.15), 0.025** |
| *6-week multivariable models include ART treatment, CD4 categorical variables, socio-economic status, race, sex, BMI for age, maternal smoke (cotinine results); only HIV variables (ART and CD4 shown) | | | | | | | | | | |
| **24-month multivariable models include ART treatment, CD4 categorical variables, socio-economic status, race, sex, BMI for age, infant smoke exposure (infant cotinine results), LRTI; only HIV variables (ART and CD4 shown) | | | | | | | | | | |
